# Supplementary material for: Emergence of changing Central-Pacific and Eastern-Pacific El Niño-Southern Oscillation in a warming climate
Source: Nat Commun. 2022 Nov 15;13:6616. doi: 10.1038/s41467-022-33930-5 (PMC9666538; doi:10.1038/s41467-022-33930-5)
Supplement: Supplementary file 1 — Supplementary Information [file 41467_2022_33930_MOESM1_ESM.pdf]

Supplementary Information for

Emergence of changing Central-Pacific and Eastern-Pacific El Niño-Southern Oscillation in a warming climate

Tao Geng[1,2], Wenju Cai[1,2,3*], Lixin Wu[1,2*], Agus Santoso[3,4,5], Guojian Wang[1,2,3], Zhao Jing[1,2], Bolan Gan[1,2], Yun Yang[6], Shujun Li[1,2], Shengpeng Wang[1,2], Zhaohui Chen[1,2], and Michael J. McPhaden[7]

[1]Pilot National Laboratory for Marine Science and Technology (Qingdao), Qingdao, China.

[2]Frontiers Science Center for Deep Ocean Multispheres and Earth System and Key Laboratory of Physical Oceanography, Ocean University of China, Qingdao, China.

[3]Centre for Southern Hemisphere Oceans Research (CSHOR), CSIRO Oceans and Atmosphere, Hobart, TAS, Australia.

[4]ARC Centre of Excellence for Climate Extremes, University of New South Wales, Sydney, NSW, Australia.

[5]Climate Change Research Centre, University of New South Wales, Sydney, NSW, Australia.

[6]College of Global Change and Earth System Science, Beijing Normal University, Beijing, China

[7]NOAA/Pacific Marine Environmental Laboratory, Seattle, WA, USA.

*Correspondence to: Wenju Cai (wenju.cai@csiro.au) or Lixin Wu (lxwu@ouc.edu.cn)

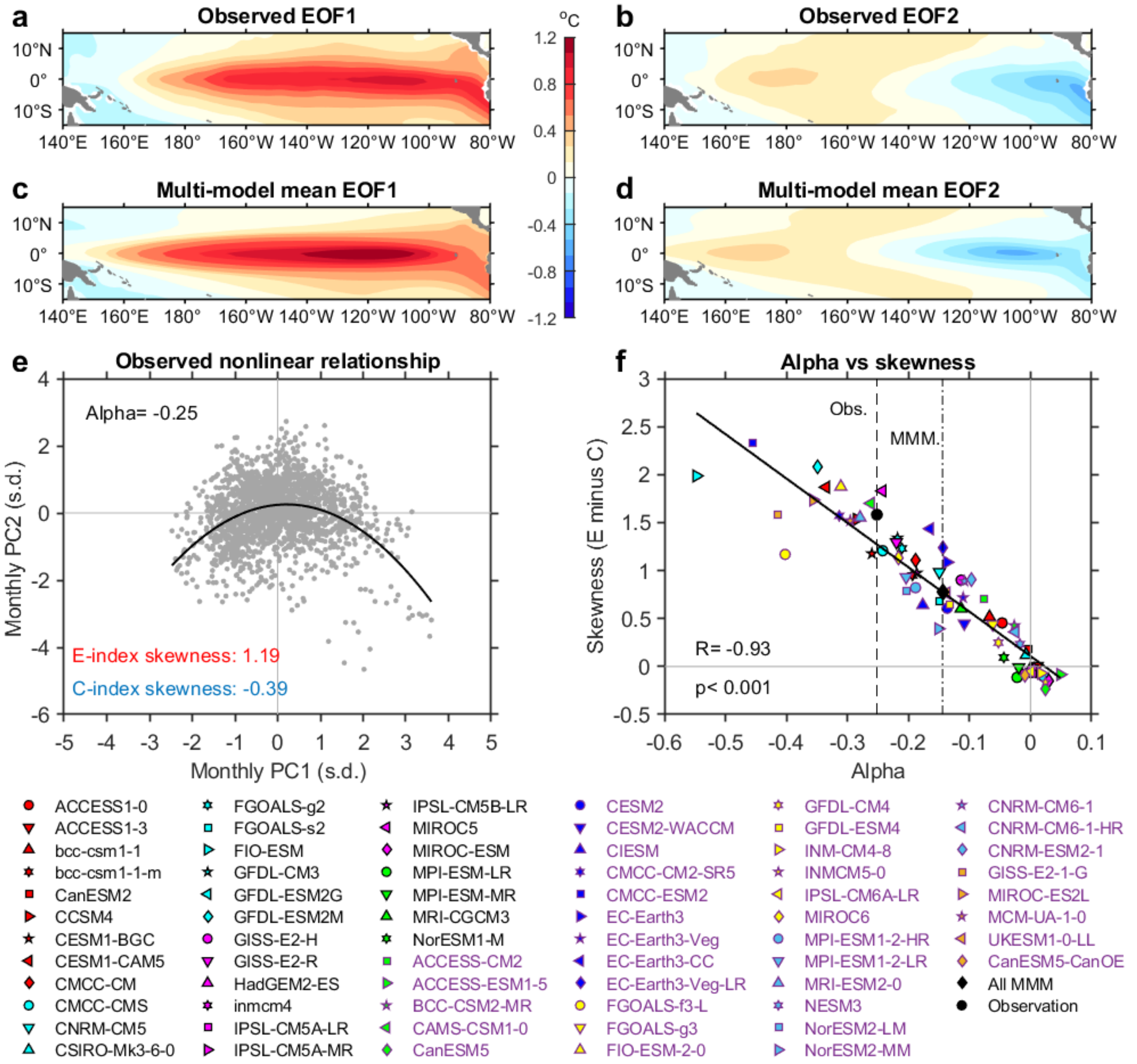

Supplementary Fig. 1 | Observed and simulated ENSO nonlinearity. **a,** EOF1 of monthly SST anomalies (°C) averaged from multiple reanalysis datasets[58-60] for the period of 1870-2019. **b,** As in **a,** but for EOF2. **c, d** Same as **a, b,** respectively, but for multi-model mean (MMM) from all models (CMIP5 in black and CMIP6 in purple) over the whole period from piControl to 2100. **e,** Nonlinear relationship between PC1 and PC2 of monthly SST anomalies (grey dots) averaged from reanalysis products. The black curve shows a quadratic fit $PC2(t)=\alpha[PC1(t)]^2+\beta PC1(t)+\gamma$, with the parameter "α" (Alpha) signifying ENSO nonlinearity. Observed values of Alpha and skewness of E-index (positive) and C-index (negative) are also indicated. **f,** Relationship between Alpha and magnitude of ENSO SST skewness, computed as skewness difference between E-index and C-index, for the whole period from piControl to 2100. The vertical dashed and dash-dotted lines indicate observed and MMM values of Alpha, respectively. The MMM value of Alpha is -0.134 (-0.153) for CMIP5 (CMIP6). Model results shown here are based on the RCP85/SSP585 scenario for a future warming climate. Note that the relationship between Alpha and ENSO SST skewness is significant in all emission scenarios.

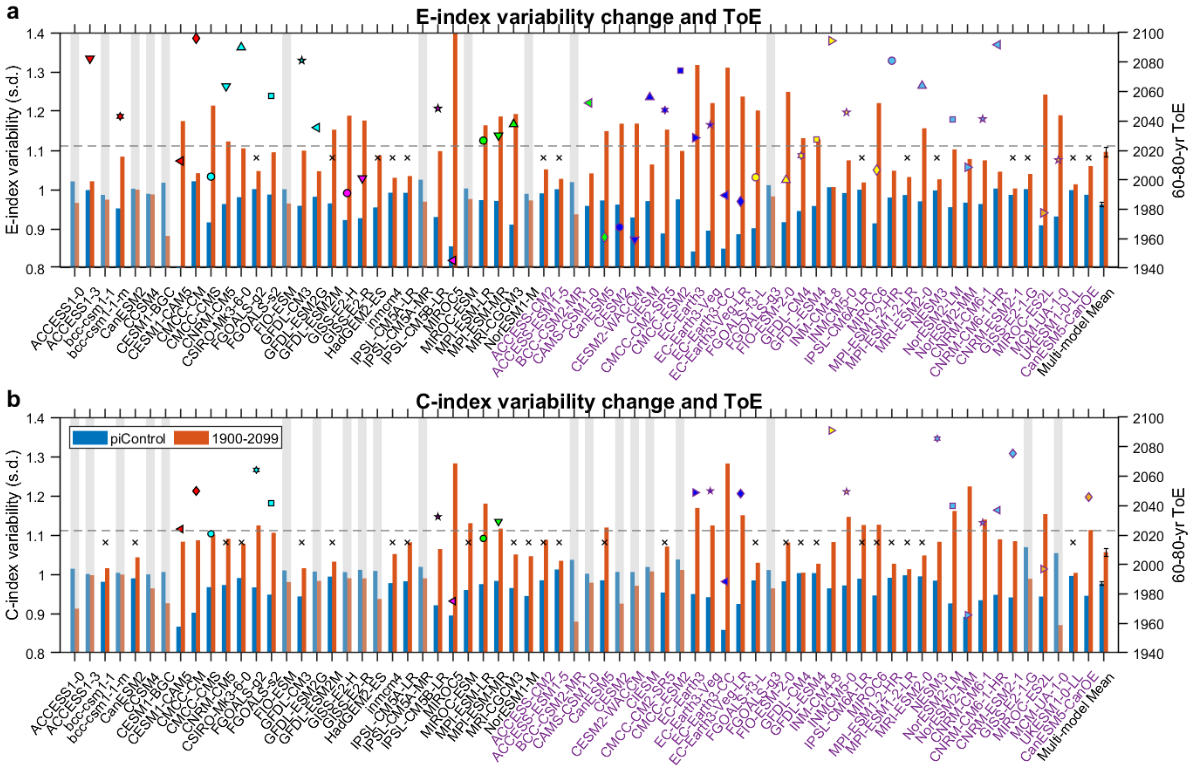

Supplementary Fig. 2 | ToE and ENSO SST variability change. a, E-index variability (left Y-axis; s.d.) over the piControl (blue bars) and 1900-2099 (red bars) periods for each and multi-model mean of all the CMIP5 and CMIP6 models. We calculate the mean level of 200-year running windowed variability of E-index in piControl. Error bars for the multi-model mean indicate one standard deviation value of 10,000 realizations using a Bootstrap test[61]. Also shown in symbols are values of the 60-80-year ToE (right Y-axis) of each individual model labelled on x-axis. The cross marker "x" indicates models that show increased E-index variability but no ToE before 2100. The horizontal dashed line denotes the year 2023. Models that do not simulate an increase in E-index variability are greyed out. CMIP5 (CMIP6) models are indicated in black (purple) and marked with black-edged (purple-edged) symbols. **b,** Same as **a**, but for C-index. Results shown here are based on the RCP85/SSP585 scenario for a future warming climate.

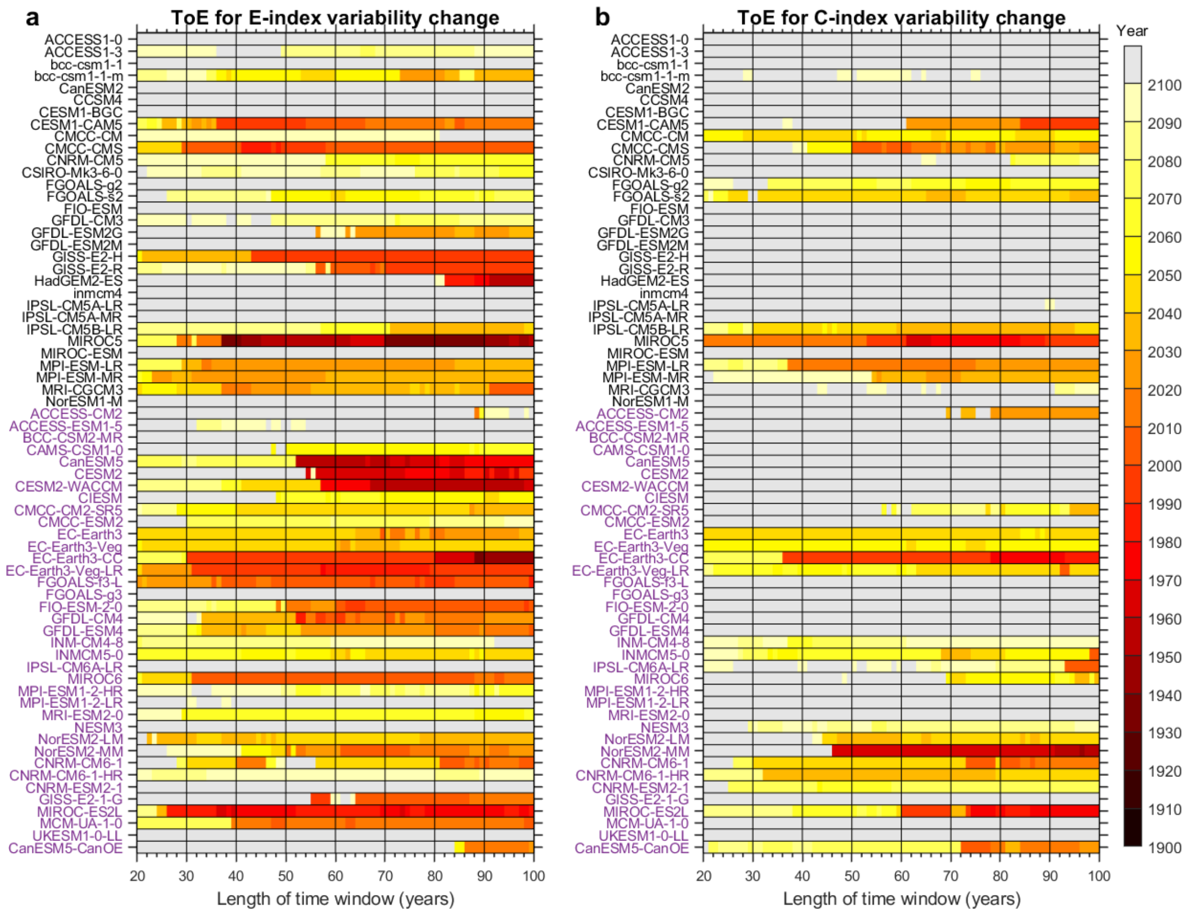

Supplementary Fig. 3 | ToE in each of the CMIP5 and CMIP6 models. a, ToE (color shading) as a function of length of time window over which signal and noise are diagnosed for E-index variability change, in each individual model (CMIP5 in black and CMIP6 in purple). **b,** Same as **a,** but for C-index. Results shown here are based on the RCP85/SSP585 scenario for a future warming climate.

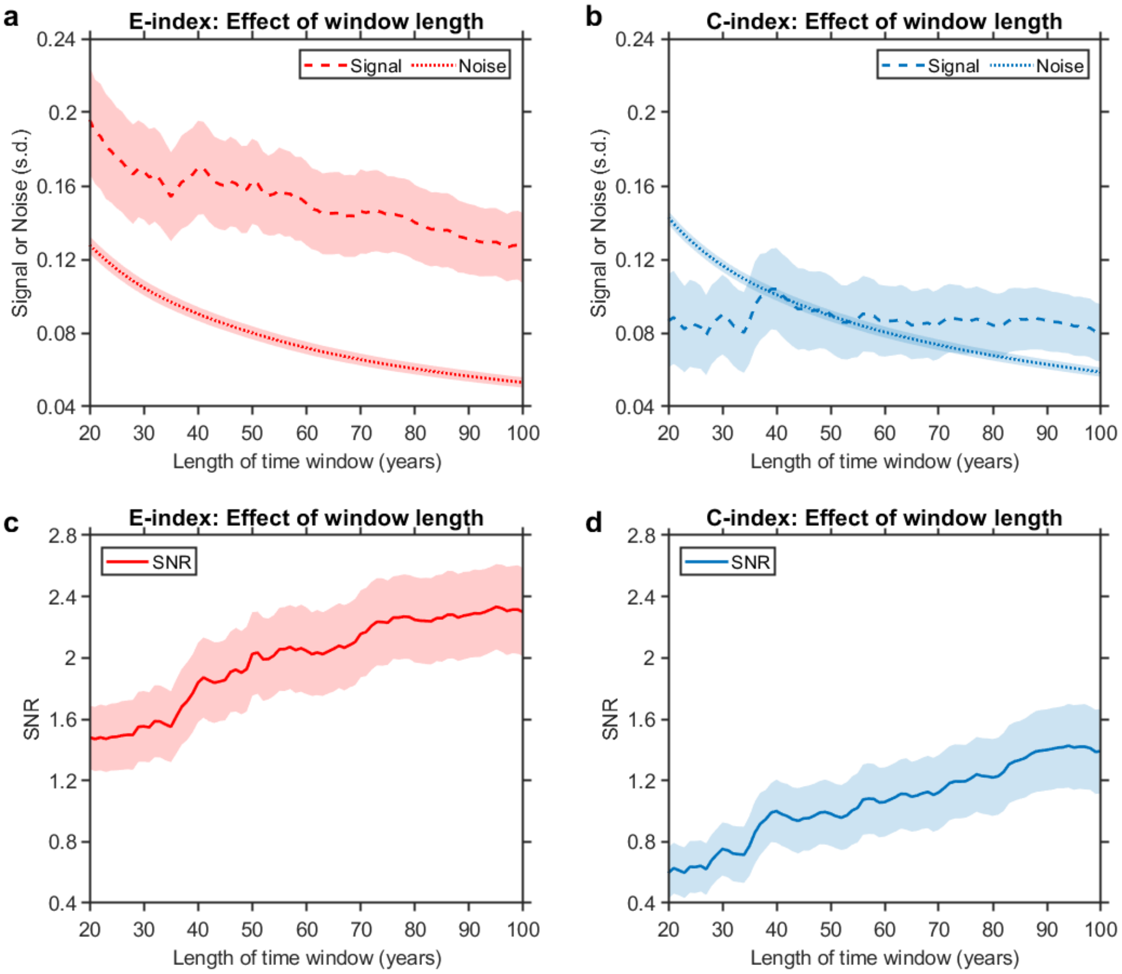

Supplementary Fig. 4 | Influence of window length on the size of signal to noise. a, Dependence of signal (red dashed line) and noise (red dotted line) upon length of time window used for diagnosing E-index. **b,** As in **a,** but for C-index. **c, d**, Same as **a, b**, respectively, but for SNR. Lines and shadings indicate multi-model mean and one standard deviation of a total of 10,000 inter-realizations using a Bootstrap method[61], respectively. Here, signal is calculated as E-index or C-index variability change from that of piControl mean in a running window that ends at 2030, and noise is defined as one standard deviation (s.d.) of the running variabilities over the piControl period. Once signal and noise are determined for a given length of time window, SNR is calculated as signal/noise. For both E-index and C-index, the longer a time window used to diagnose signal, the better it is to maximize the effect of climate change signal and minimize the influence of internal variability of the climate system, thus leading to a larger SNR. Results shown here are based on the RCP85/SSP585 for a future warming scenario. The 47 models that show 60-80-year ToE of either E-index or C-index before 2100 are used here.

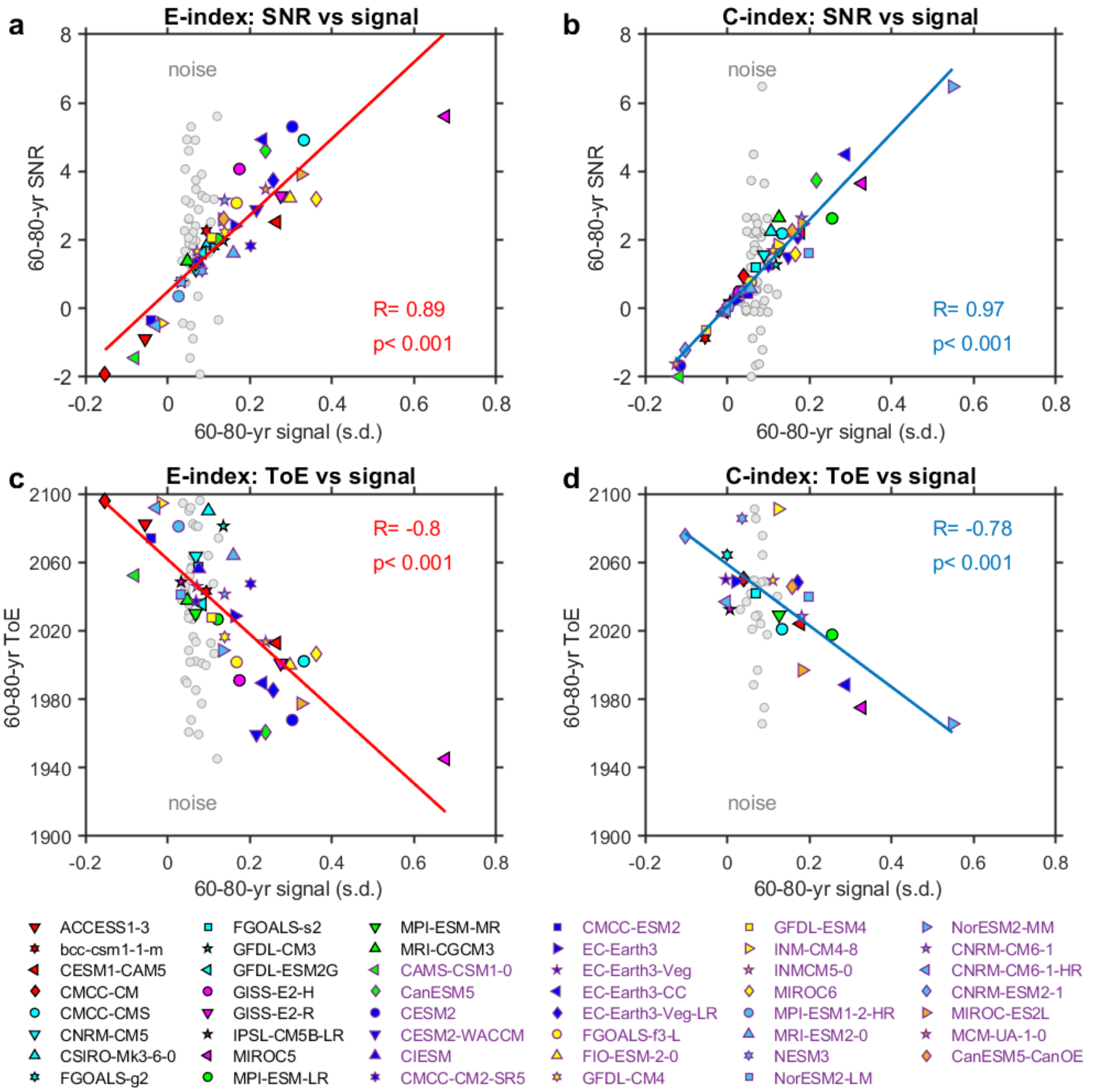

Supplementary. Fig. 5 | ToE dominated by signal of SST variability change. a, b, Inter-model relationship between 60-80-year Signal-to-Noise-Ratio (SNR) and signal for (**a**) E-index and (**b**) C-index, in 47 models (symbols; CMIP5 in black and CMIP6 in purple) that show ToE of either E-index or C-index before 2100. For comparison, noise levels are also shown in grey filled circles. Here we focus on the 60-80-year window. Signal is calculated as the 1960-2030 E-index or C-index variability change from the mean 60-80-year windowed variability in piControl, and noise is defined as one standard deviation (s.d.) of the 60-80-year windowed variability in piControl. **c, d,** As in **a, b,** respectively, but for ToE and signal. Linear fits (solid lines) are displayed in together with correlation coefficient R and corresponding p value. Results shown here are based on the RCP85/SSP585 scenario for a future warming climate.

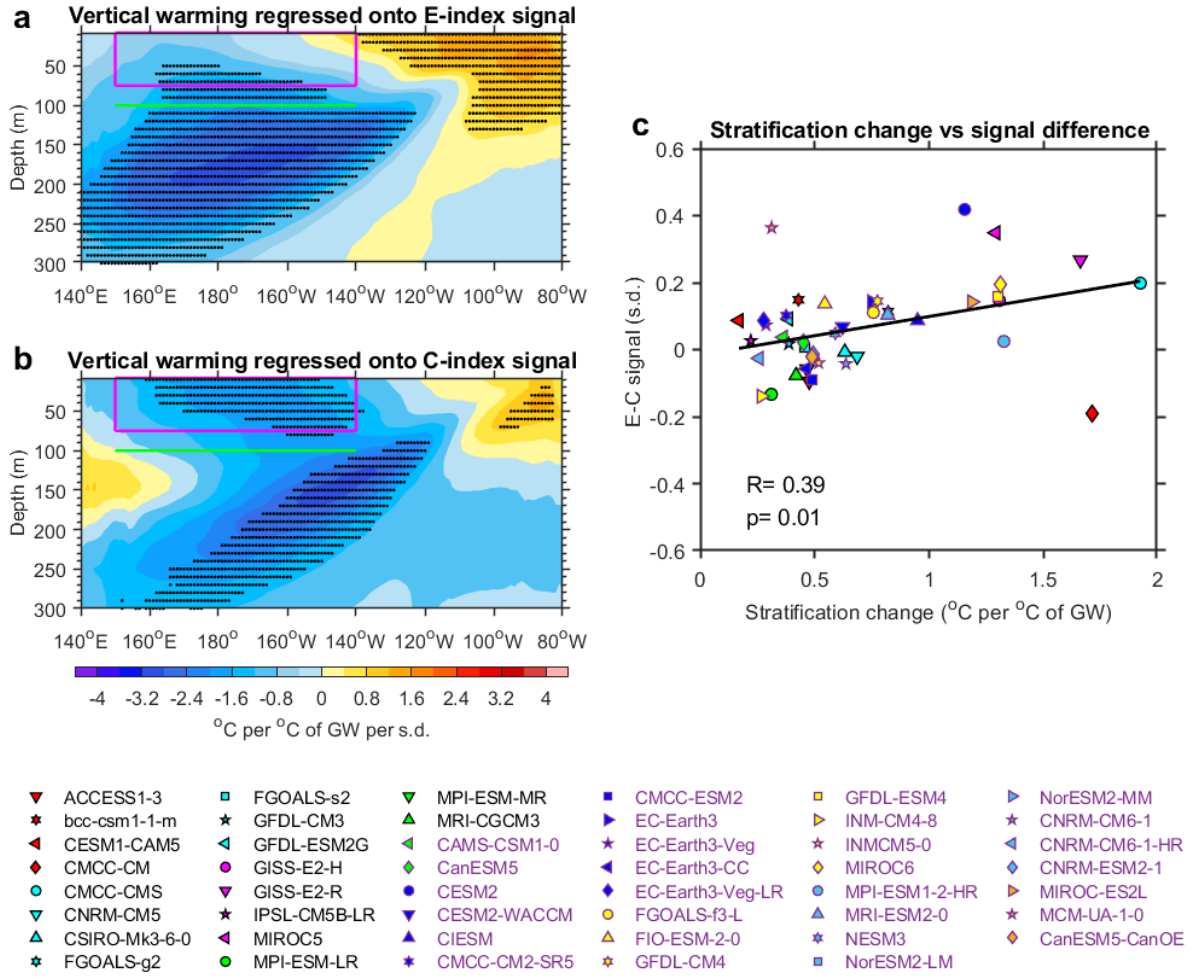

Supplementary Fig. 6. | Influence of ocean stratification change on signal of ENSO SST variability change. a, Inter-model regression of mean equatorial (average over 5°S-5°N) upper-ocean temperature change onto 60-80-year signal of E-index. Temperature changes are taken as the mean difference between 1960-2030 and 1850-1899, a surrogate period of piControl climatology, and scaled by corresponding global-mean SST increase (i.e., global warming, GW) between the two periods in each model. **b,** As in **a,** but for C-index. Black stippling in **a, b** indicates statistical significance above the 95% confidence level based on a two-tailed Student's t test. **c**, Inter-model relationship between change (1960-2030 minus 1850-1899, scaled by GW) in ocean stratification and E-minus-C signal difference. The ocean stratification is calculated as the difference between the mean temperature over the upper 75m (purple box in **a, b**) and the temperature at 100m (green line in **a, b**), both averaged over the longitudinal range of 150°E-140°W following a previous study[23]. A linear fit (solid line) is displayed together with correlation coefficient R and corresponding p value. Using the whole equatorial Pacific (120°E-80°W, 5°S-5°N) to calculate the stratification change, the inter-model correlation between stratification change and E-minus-C signal difference is r=0.42. Results shown here are based on the RCP85/SSP585 scenario for a future warming climate. The 47 models that show 60-80-year ToE of either E-index or C-index before 2100 are used.

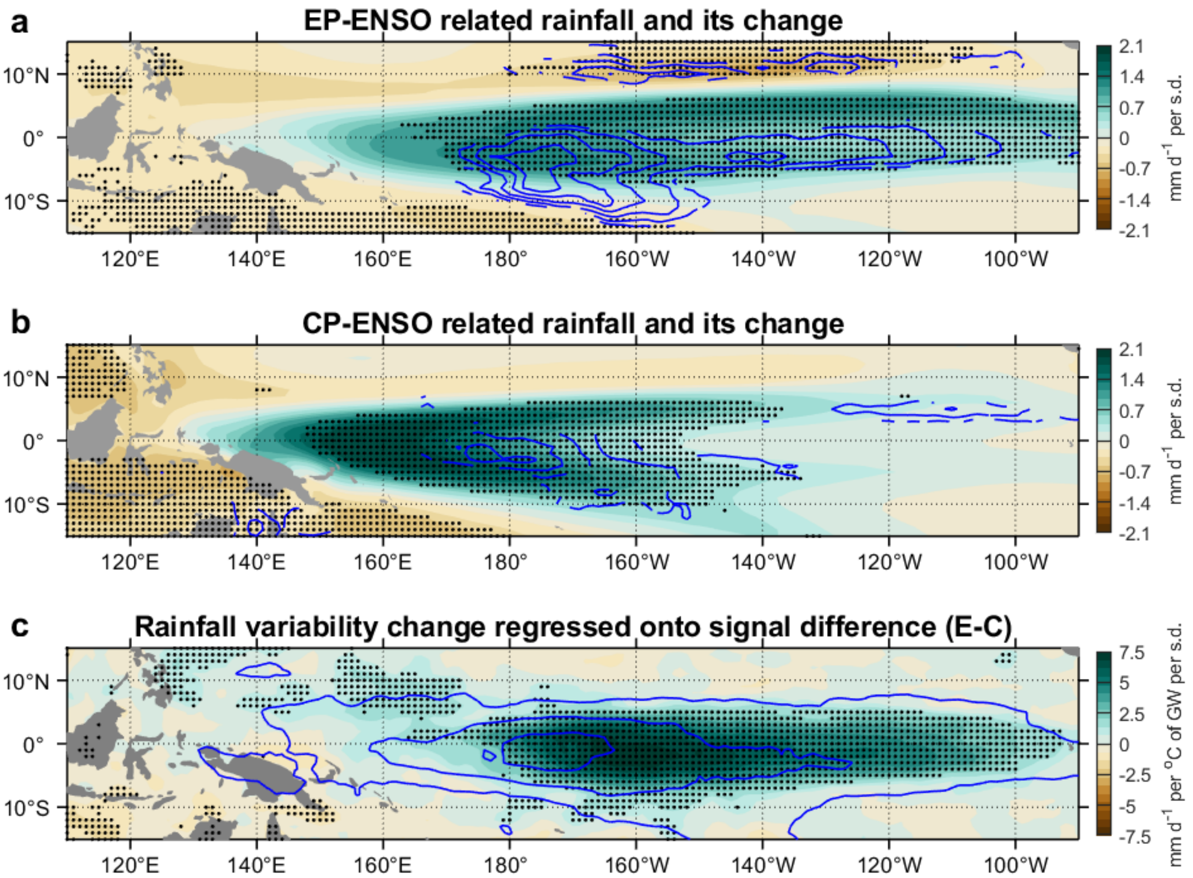

Supplementary Fig. 7. | Rainfall changes associated with EP-ENSO and CP-ENSO. a, Multi-model mean pattern of EP-ENSO induced rainfall (shading) by regression of grid-point rainfall anomalies onto E-index over the whole period. Dots denote that more than 90% of models showing same-signed response. Contours (positive in blue; with an interval of 0.05 mm d^{-1} per s.d. per °C of GW) denote multi-model mean change (1960-2030 minus piControl) of regression coefficients that are significant above the 95% level based on a Bootstrap test[61]. **b,** As in **a,** but for C-index. **c,** Inter-model regression of grid-point rainfall variability change (1960-2030 minus piControl, scaled by per °C of global warming) onto 60-80-year E-minus-C signal difference (s.d.). Black stippling indicates statistical significance above the 95% confidence level based on a two-tailed Student's t test. Contours (positive in blue; with an interval of 0.4 mm d^{-1} per °C of GW) denote multi-model mean changes (1960-2030 minus piControl) of rainfall variability changes that are significant above the 95% confidence level based on a Bootstrap test[61]. Results shown here are based on the RCP85/SSP585 scenario for a future warming climate. The 47 models that show 60-80-year ToE of either E-index or C-index before 2100 are used.

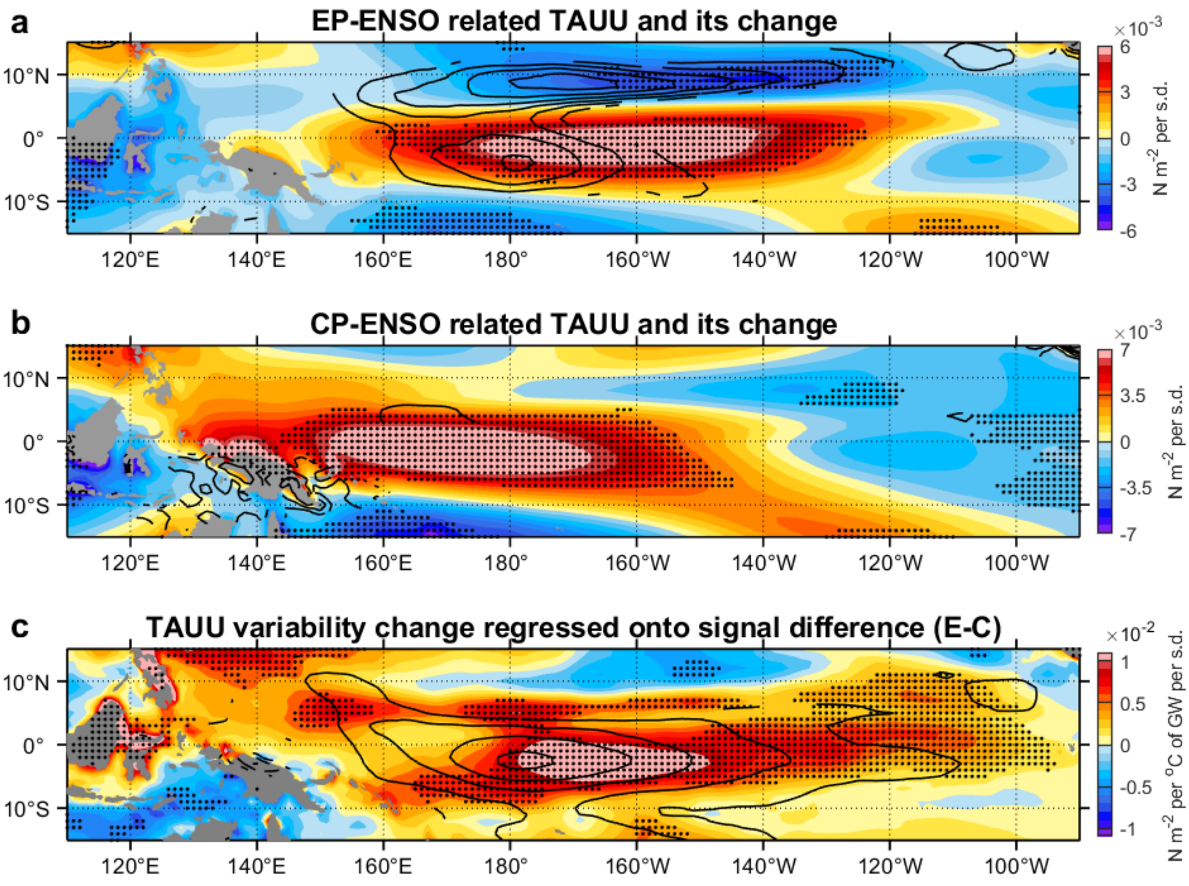

Supplementary Fig. 8. | Zonal wind changes associated with EP-ENSO and CP-ENSO. As in Supplementary Fig. 7, but for surface zonal wind stress (TAUU). **a,** Multi-model mean pattern of EP-ENSO induced TAUU (shading) by regression of grid-point TAUU anomalies onto E-index over the whole period. Dots denote that more than 90% of models showing same-signed response. Contours (positive in black; with an interval of 0.4×10^{-3} N m^{-2} per s.d. per °C of global warming) denote multi-model mean changes (1960-2030 minus piControl) of corresponding regression coefficients that are significant above the 95% confidence level based on a Bootstrap test[61]. **b,** As in **a,** but for C-index. **c,** Inter-model regression of grid-point TAUU variability change (1960-2030 minus piControl, scaled by per °C of global warming) onto 60-80-year E-minus-C signal difference (s.d.). Black stippling indicates statistical significance above the 95% confidence level based on a two-tailed Student's t test. Contours (positive in black; with an interval of 0.8×10^{-3} N m^{-2} per °C of global warming) denote multi-model mean changes (1960-2030 minus piControl) of TAUU variability changes that are significant above the 95% confidence level based on a Bootstrap test[61]. Results shown here are based on the RCP85/SSP585 scenario for a future warming climate. The 47 models that show 60-80-year ToE of either E-index or C-index before 2100 are used.

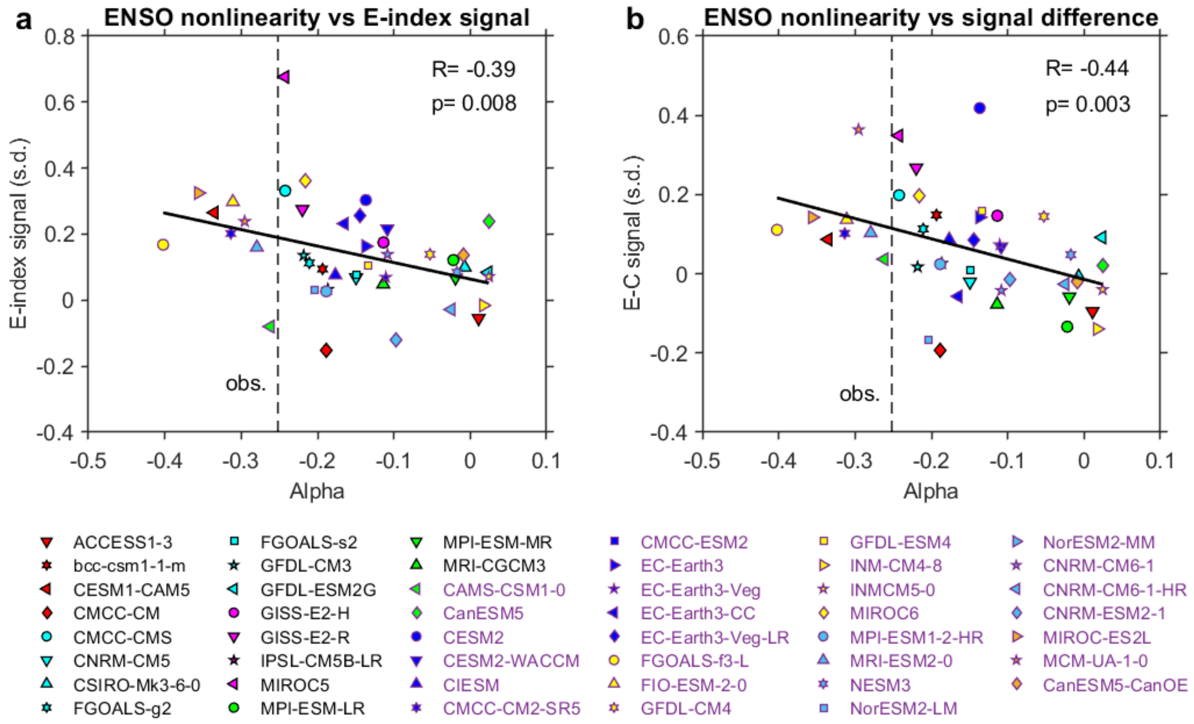

Supplementary Fig. 9. | Influence of ENSO nonlinearity on signal of ENSO SST variability change. **a**, Inter-model relationship between ENSO nonlinearity, measured by Alpha in the whole period (see Methods), and 60-80-year E-index signal (s.d.). **b,** As in **a,** but for inter-model relationship between Alpha and E-minus-C signal difference (s.d.). The vertical dashed lines in **a, b** indicate multi-product averaged value of Alpha from observations. The linear fits (solid lines) are displayed in **a, b** together with correlation coefficient R and corresponding p value. Results shown here are based on the RCP85/SSP585 scenario for a future warming climate, focusing on the 60-80-year window. The 47 models that show 60-80-year ToE of either E-index or C-index before 2100 are used.

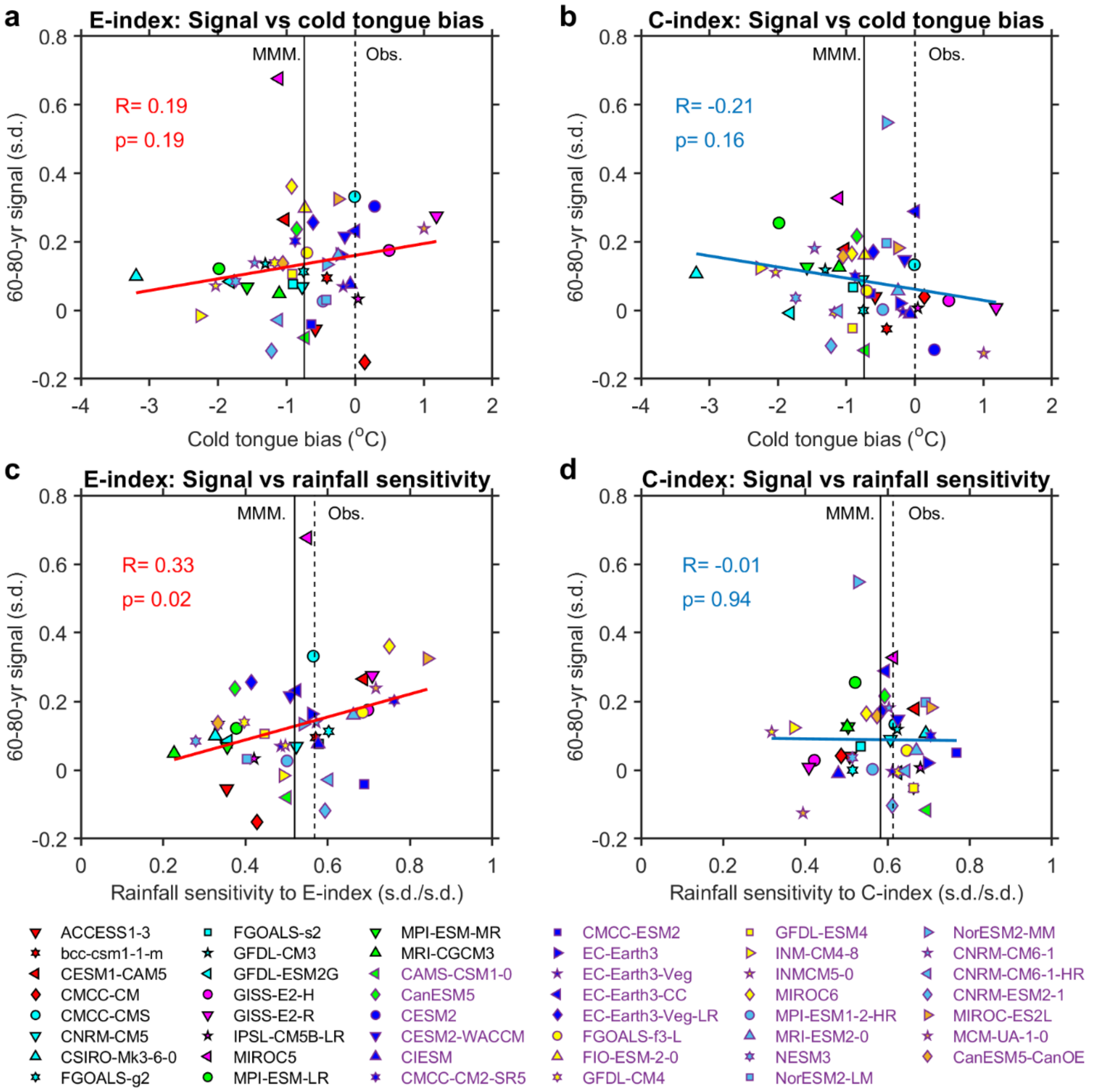

Supplementary Fig. 10 | Influence of model bias on signal of ENSO SST variability change. **a**, Inter-model relationship between cold tongue bias (°C), measured by model-observation difference in climatological SST over the equatorial Pacific region (160°E-100°W, 2°S-2°N) in the historical period of 1900-1999, and 60-80-year E-index signal (s.d.). The vertical dashed and solid lines indicate observed (obs) and multi-model mean (MMM) values of the bias, respectively. The linear fit (red solid line) is displayed together with correlation coefficient R and corresponding p value. **b**, As in **a**, but for C-index. **c, d,** Same as **a, b,** respectively, but for inter-model relationship between rainfall sensitivity to SST and 60-80-year ENSO SST variability signal. The rainfall sensitivity is calculated for each individual model as the regression coefficient of normalized (5°S-5°N average) rainfall anomalies onto E-index or C-index at corresponding maximum response centres in 1900-1999. Observed rainfall is from GPCP[62] and SST is from HadISST[57]. Results shown here are based on the RCP85/SSP585 scenario for a future warming climate, focusing on the 60-80-year window. The 47 models are used that show a ToE of either E-index or C-index before 2100 using the 60-80-year window.

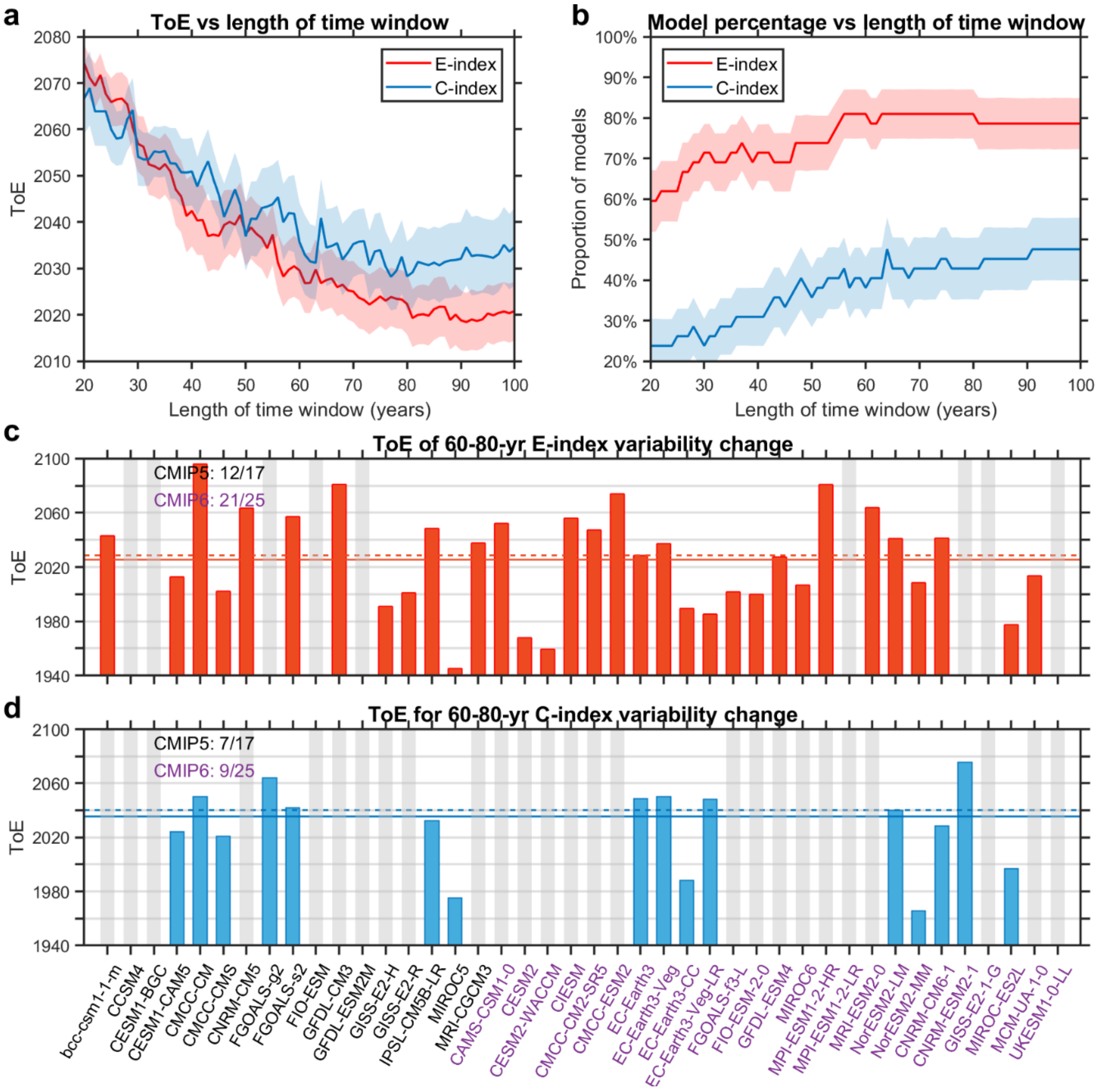

Supplementary Fig. 11 | Sensitivity to model selection. Same as Fig. 2, but for 42 selected models of which Alpha is at least one third of the observed value. **a,** Dependence of ToE upon length of time window over which signal and noise are diagnosed for E-index (red) and C-index (blue). **b,** As in **a**, but for proportion (in percentage) of models that show emergence of E-index (red) and C-index (blue) before 2100. Solid lines and shadings indicate multi-model mean and one standard deviation of a total of 10,000 inter-realizations, respectively, based on a Bootstrap method (see Methods). **c,** ToE diagnosed from a 60-80-year sliding window for E-index variability in 42 selected models. Only models that show ToE spanning the whole 60-80-year window are indicated by red bars, otherwise they are greyed out. Multi-model mean and median values are indicated by the horizontal solid and dashed lines, respectively. Numbers on the upper left denote the number of models showing ToE in the CMIP5 (black) or CMIP6 (purple) multi-model ensemble. **d,** As in **c,** but for C-index. Results shown here are based on the RCP85/SSP585 scenario for a future warming climate. As shown in **a**, the uncertainties of the two types of ENSO overlap to some extent, indicating that the earlier ToE of EP-ENSO than CP-ENSO includes some uncertainties in the 42 models. Nevertheless, our key results still hold, including ToE decreasing with increasing lengths of time window and EP-ENSO emerging earlier than CP-ENSO. For example, in the range of 60-80 years, 78.57% (33 out of

42) of all models generate a ToE of EP-ENSO with a MMM value of ~2026, whereas only 38.10% (16 out of 42) of models generate a ToE of CP-ENSO with a MMM value of ~2035. Using other thresholds (0 or 1/2 of observed amplitude) of Alpha to select models yields similar results.

Supplementary Table 1 | Details of Models. CMIP5 and CMIP6 models used in the present study. Both Alpha and skewness of E-index and C-index are calculated from the concatenated SST anomalies from piControl to 2100, using RCP85/SSP585 as a future warming scenario. "*" denotes that ocean temperature data are not available for the assessment of vertical temperature change.

Ensemble member	Model name	piControl years	Alpha	E-index skewness	C-index skewness
r1i1p1	ACCESS1-0	500	-0.047	0.068	-0.386
r1i1p1	ACCESS1-3	500	0.011	-0.020	-0.025
r1i1p1	bcc-csm1-1	500	-0.067	0.104	-0.412
r1i1p1	bcc-sm1-1-m	400	-0.193	0.466	-0.483
r1i1p1	CanESM2	996	-0.003	-0.179	-0.364
r1i1p1	CCSM4	1051	-0.285	0.877	-0.655
r1i1p1	CESM1-BGC	500	-0.260	0.612	-0.565
r1i1p1	CESM1-CAM5	319	-0.335	1.230	-0.639
r1i1p1	CMCC-CM	330	-0.188	0.785	-0.328
r1i1p1	CMCC-CMS	500	-0.242	0.784	-0.423
r1i1p1	CNRM-CM5	850	-0.150	0.457	-0.526
r1i1p1	CSIRO-Mk3-6-0	500	-0.007	0.075	-0.046
r1i1p1	FGOALS-g2	700	-0.211	0.406	-0.823
r1i1p1	FGOALS-s2	500	-0.148	0.339	-0.345
r1i1p1	FIO-ESM	800	-0.547	1.186	-0.800
r1i1p1	GFDL-CM3	800	-0.218	0.462	-0.878
r1i1p1	GFDL-ESM2G	500	0.023	-0.158	-0.031
r1i1p1	GFDL-ESM2M	500	-0.350	1.487	-0.597
r1i1p1	GISS-E2-H	540	-0.114	0.450	-0.451
r1i1p1	GISS-E2-R	550	-0.220	0.509	-0.786
r1i1p1	HadGEM2-ES	576	-0.001	-0.157	-0.127
r1i1p1	inmcm4	500	0.005	0.030	0.041
r1i1p1	IPSL-CM5A-LR	1000	0.004	-0.106	-0.024
r1i1p1	IPSL-CM5A-MR	300	-0.017	0.032	0.089
r1i1p1	IPSL-CM5B-LR	300	-0.186	0.597	-0.378
r1i1p1	MIROC5	870	-0.243	1.292	-0.538
r1i1p1	MIROC-ESM	680	0.030	-0.071	0.083
r1i1p1	MPI-ESM-LR	1000	-0.022	-0.130	-0.016
r1i1p1	MPI-ESM-MR	1000	-0.019	-0.073	-0.065
r1i1p1	MRI-CGCM3	500	-0.114	0.312	-0.291
r1i1p1	NorESM1-M	501	-0.044	0.041	-0.053
r1i1p1f1	ACCESS-CM2	500	-0.076	0.236	-0.464
r1i1p1f1	ACCESS-ESM1-5	1000	0.049	-0.165	-0.082
r1i1p1f1	BCC-CSM2-MR	600	-0.027	-0.035	-0.463
r1i1p1f1	CAMS-CSM1-0	500	-0.262	0.893	-0.807

r1i1p1f1	CanESM5	1000	0.024	-0.201	0.030
r1i1p1f1	CESM2	1200	-0.137	0.111	-0.495
r1i1p1f1	CESM2-WACCM	499	-0.109	0.061	-0.386
r1i1p1f1	CIESM	500	-0.177	0.272	-0.369
r1i1p1f1	CMCC-CM2-SR5	500	-0.314	1.083	-0.487
r1i1p1f1	CMCC-ESM2	500	-0.455	1.556	-0.779
r1i1p1f1	EC-Earth3	501	-0.137	0.728	-0.359
r1i1p1f1	EC-Earth3-Veg	499	-0.111	0.394	-0.324
r1i1p1f1	EC-Earth3-CC	505	-0.165	0.818	-0.618
r1i1p1f1	EC-Earth3-Veg-LR	501	-0.144	0.792	-0.449
r1i1p1f1	FGOALS-f3-L	500	-0.402	0.504	-0.664
r1i1p1f1	FGOALS-g3	700	-0.062	-0.075	-0.514
r1i1p1f1	FIO-ESM-2-0	575	-0.311	1.051	-0.823
r1i1p1f1	GFDL-CM4	500	-0.053	0.065	-0.184
r1i1p1f1	GFDL-ESM4	500	-0.133	0.271	-0.376
r1i1p1f1	INM-CM4-8	531	0.017	-0.171	-0.101
r1i1p1f1	INM-CM5-0	1201	0.024	-0.219	-0.057
r1i1p1f1	IPSL-CM6A-LR	2000	0.001	-0.171	-0.119
r1i1p1f1	MIROC6	800	-0.216	0.881	-0.252
r1i1p1f1	MPI-ESM1-2-HR	500	-0.189	0.418	-0.403
r1i1p1f1	MPI-ESM1-2-LR	1000	-0.204	0.545	-0.386
r1i1p1f1	MRI-ESM2-0	701	-0.279	1.017	-0.534
r1i1p1f1	NESM3	500	-0.017	0.084	-0.148
r1i1p1f1	NorESM2-LM*	501	-0.204	0.206	-0.576
r1i1p1f1	NorESM2-MM*	500	-0.151	0.087	-0.306
r1i1p1f2	CNRM-CM6-1	500	-0.108	0.103	-0.785
r1i1p1f2	CNRM-CM6-1-HR	300	-0.025	0.077	-0.284
r1i1p1f2	CNRM-ESM2-1	500	-0.097	-0.007	-0.915
r1i1p1f2	GISS-E2-1-G	345	-0.415	0.780	-0.803
r1i1p1f2	MIROC-ES2L	500	-0.356	1.098	-0.637
r1i1p1f2	MCM-UA-1-0	500	-0.295	1.052	-0.468
r1i1p1f2	UKESM1-0-LL	1100	-0.138	0.230	-0.536
r1i1p2f1	CanESM5-CanOE	501	-0.009	-0.189	-0.096

Supplementary Table 2 | ToE sensitivity to different thresholds and emission scenarios. Shown are multi-model ensemble mean plus/minus inter-model spread (one standard deviation from a Bootstrap test[61]) estimates of 70-year ENSO ToE with E-index and C-index, using different thresholds, 2 standard deviations (2 STD) or maximum (MAX) values of 70-year windowed variability in piControl, for signal detection and in different warming scenarios. Also shown are proportion (%) of models that show ToE before 2100. Numbers with (without) asterisks indicate results based on all CMIP models (CMIP6 models only).

Emission scenario	E-index		C-index	
	2 STD	MAX	2 STD	MAX
RCP85 or SSP585	*2030±6.1<br>*65%±5.8%	*2033±6.3<br>*63%±5.8%	*2043±6.2<br>*38%±5.9%	*2037±7.3<br>*37%±5.9%
	2024±7.4<br>73%±7.5%	2027±7.4<br>73%±7.3%	2040±8.0<br>46%±8.2%	2038±10.2<br>43%±8.1%
SSP370	2026±9.5<br>61%±8.7%	2026±9.7%<br>61%±8.8%	2056±10.3<br>45%±8.9%	2055±12.4<br>35%±8.6%
RCP45 or SSP245	*2032±7.2<br>*52%±6.1%	*2033±7.8<br>*49%±6.1%	*2047±8.9<br>*31%±5.7%	*2050±8.5<br>*25%±5.4%
	2033±8.9<br>64%±8.1%	2032±9.8<br>56%±8.2%	2047±11.1<br>39%±8.0%	2057±10.5<br>33%±7.8%
RCP26 or SSP26	*2033±8.4<br>*45%±6.5%	*2029±8.7<br>*41%±6.5%	*2056±9.4<br>*31%±6.0%	*2058±10.2<br>*29%±6.0%
	2035±10.6<br>52%±9.7%	2035±10.6<br>52%±9.6%	2057±10.4<br>44%±9.5%	2058±11.6<br>44%±9.7%

Reference

62. Adler, R. F., et. Al. The Global Precipitation Climatology Project (GPCP) monthly analysis (new version 2.3) and a review of 2017 global precipitation. *Atmosphere*, **9**, 138 (2018).
